# Accurate diagnosis of bronchopulmonary *Talaromyces marneffei* infection in an anti-IFN-γ autoantibodies positive patient assisted by endobronchial ultrasound-guided TBNA and mNGS: a case report

**DOI:** 10.3389/fcimb.2023.1186335

**Published:** 2023-10-04

**Authors:** Guirong Chen, Nan Ma, Donglan Zhu, Huaihai Zhou, Qiumei Liang, Jianfeng Meng, Yin Shen, Hang Liu, Liu Liu, Zhiyi He, Zhiqiang Qin

**Affiliations:** ^1^ Department of Pulmonary and Critical Care Medicine, The People’s Hospital of Guangxi Zhuang Autonomous Region, Nanning, China; ^2^ Department of International Medicine, The People’s Hospital of Guangxi Zhuang Autonomous Region, Nanning, China; ^3^ Department of Pulmonary and Critical Care Medicine, The First Affiliated Hospital of Guangxi Medical University, Nanning, China; ^4^ Department of Pulmonary Medicine, Foresea Life Insurance Guangxi Hospital, Nanning, China

**Keywords:** anti-interferon-gamma autoantibodies, endobronchial ultrasound, lung, mediastinal lymph node, metagenomic next-generation sequencing, *Talaromyces marneffei*

## Abstract

Rationale: *T. marneffei* is opportunistic and dimorphic fungus, which can cause systemic mycosis in human beings. It’s being difficult to obtain histopathological or microbiological evidence in *T. marneffei* infection. We reported a rare non-HIV case of *T. marneffei* infection of bronchopulmonary and mediastinal lymph nodes which was diagnosed by EBUS-TBNA combined with mNGS. The high titer of anti-IFN-γ autoantibodies in serum was probably the cause of *T. marneffei* infection,which has yet to be fully known. Patient concerns: A 56-year-old Chinese man presented with a 5-month history of intermittent low or high fever and dry cough, followed by fatigue, night sweating, and chest pain when coughing. A large hilar lesion in the left lung and multiple mediastinal lymph node enlargements were found on his chest CT scan. Diagnoses: The patient received EBUS-TBNA of hilar tissue and lymph node biopsy for mNGS at the second Ultrasonic bronchoscopy. No fungal hyphae or spores were found in the histopathology. There were high sequencing reads of *T. marneffei* in samples of lymph node fluid and bronchogenesis tissue detected by mNGS. His plasma anti-IFN-γ autoantibodies level was positive with a high titer at 1:2500↑. Intervention: The patient went through atrial fibrillation at the first dose of amphotericin B liposomes and treated with voriconazole later. Outcomes: His fever, cough and dyspnea quickly disappeared since the fourth day of treatment. After six months, there was not any focus in his chest CT scans. But his plasma anti-IFN-γ autoantibodies remained unchanged. Lessons: Complementing the traditional laboratory and bronchoscopy, mNGS combined with EBUS-TBNA facilitate rapid and precise diagnosis of bronchopulmonary mediastinal lymph nodes *T. marneffei* infection. Clinicians should be aware of anti-INF-γ autoantibodies in opportunistic infections of non-HIV patients.

## 1 Introduction

*Talaromyces marneffei (*T. marneffei*)* is a pathogenic, thermal dimorphic fungus that has mostly been reported in areas of endemicity in Southeast Asia, including Thailand, India, Vietnam, and China (Cao et al., 2019). *T. marneffei* can cause disseminated infections and invade multiple organs, such as the lungs, blood, bone marrow, central nervous system, and skin (Cao et al., 2019). Disseminated *T. marneffei* is known to predominantly occur in immunocompromised patients, such as HIV patients (Limper et al., 2017) and patients receiving monoclonal antibody treatment (Chan et al., 2015) but is seldom reported in immunocompetent patients. However, clinicians intermittently encounter HIV-negative *T. marneffei* infection case (Chen et al., 2021; Zhang et al., 2021). Patients present different clinical manifestations based on infection sites and the degree of infection (Cao et al., 2019).

Bronchopulmonary *T. marneffei* diseases may cause fever, cough, expectoration, chest pain, dyspnea, and other clinical symptoms, and signs (Cao et al., 2019). Patch, mass, cavity, pleural effusion, and hilar and mediastinal lymph node enlargement can be found on chest imaging (Chen et al., 2021). Since the clinical manifestation is not specific, the disease has a great chance of being erroneously diagnosed as lung cancer, tuberculosis, lymphoma, etc. The case we discussed here was initially misdiagnosed as cancer. The diagnosis of bronchopulmonary *T. marneffei* disease requires positive microbial evidences and/or histopathological *T. marneffei* infection evidence. However, the difficulty in obtaining the target tissue and the low positive rate of microbial culture and histological analysis lead to the delay of diagnosis or even misdiagnosis. Patients who fail to receive timely and accurate treatment progress rapidly and endanger their lives (Cao et al., 2019; Pruksaphon et al., 2022).

Bronchopulmonary mediastinal lymph node *T. marneffei* infection often has lung shadow and mediastinal lymph node enlargement. Bronchoscopy combined with EBUS-TBNA can obtain the focus tissue accurately and safely (Dhooria et al., 2015). At the same time, the wide application of mNGS in the clinic is expected to make up for the deficiency of the low positive rate of culture and histopathology analysis (Tsang et al., 2021). This article reports bronchopulmonary *T. marneffei* disease diagnosed by ultrasound bronchoscope-guided lung and lymph node biopsy combined with mNGS. The patient’s diagnosis was corrected in time, which pointed out the right direction for his following treatment. Since *T. marneffei* infection is most often seen in opportunistic infections, the real immune status of patients is worthy of further clinical exploration. We found that the patient’s immunosuppression may have resulted from a high titer of anti-IFN-γ autoantibodies. Although bronchopulmonary *T. marneffei* infection was relieved, his serum anti-IFN-γ autoantibodies level remained elevated.

## 2 Case presentations

**Figure 1:**
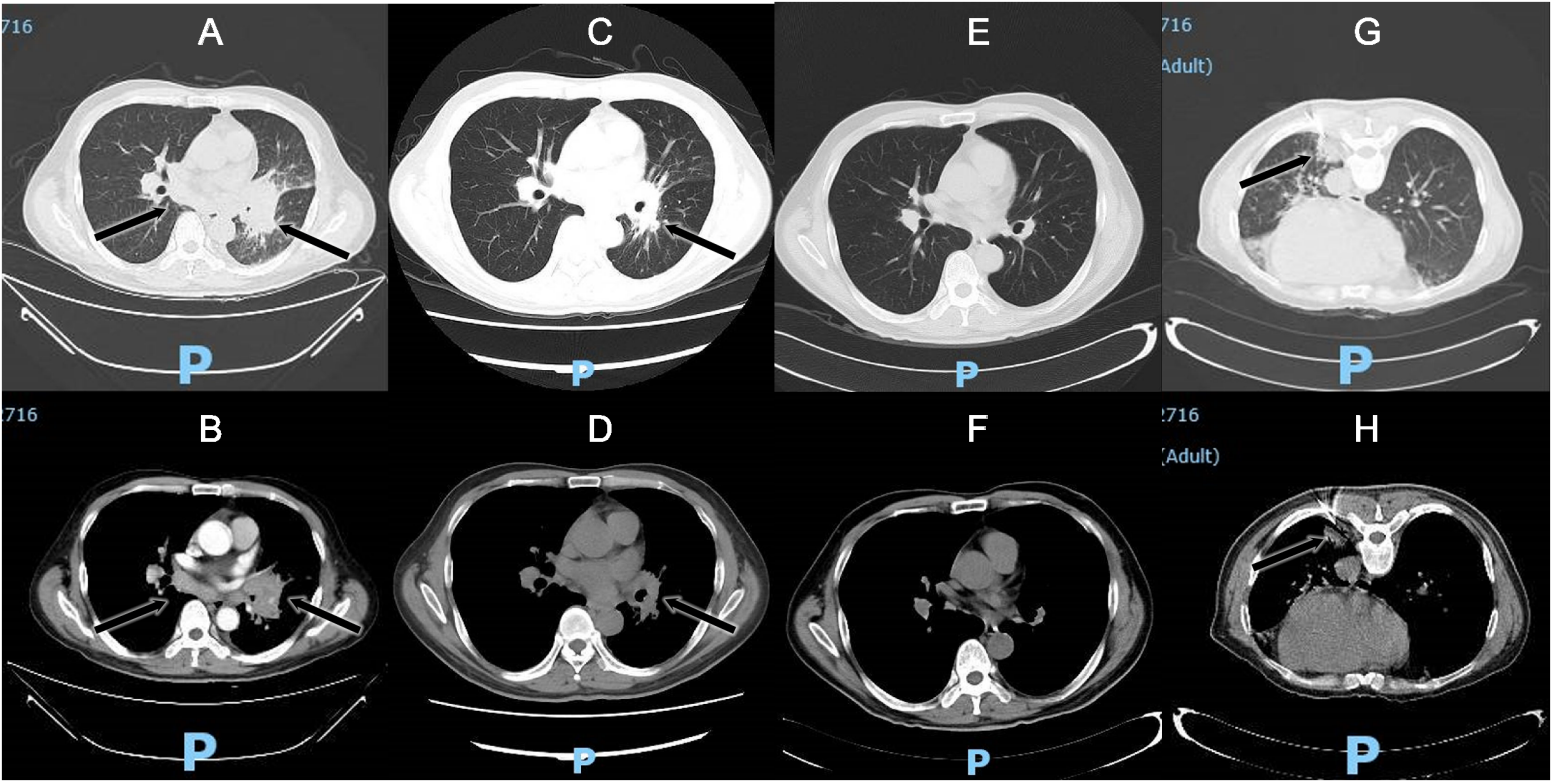
Chest CT scan: subcarinal lymphadenopathy, irregular soft tissue mass in the left hilum and uneven enhancement on the enhanced scan **(A)** Lung window; **(B)** Enhanced scanning); Lesions were significantly absorbed on chest CT scans after 16 days of voriconazole treatment **(C)** Lung window; **(D)** Mediastinal window); Lesions disappeared on chest CT scans after 6 months of voriconazole treatment **(E)** Lung window; **(F)** Mediastinal window); CT-guided percutaneous puncture of the left lower lung biopsy **(G)** Lung window; **(H)** Mediastinal window).

A 56-year-old Chinese man presented with a 5-month history of intermittent low or high fever (Tmax 39.4°C) and dry cough, followed by fatigue, night sweating, and chest pain when coughing. The local hospital suspected primary bronchial lung cancer complicated with obstructive pneumonia; therefore, traditional bronchoscopy was performed but could not detect tumors or clear pathogenic microorganisms. Then, the patient received empirical antibacterial treatment with piperacillin-tazobactam, levofloxacin, and prescribed azithromycin. However, no improvement was observed after these treatments. Later, the patient was transferred to provincial general hospital on Jan 21st, 2022. The patient reported an hepatitis B virus(HBV) carrier and gallstones without digestive symptoms as a part of his medical history and denied any history of tuberculosis infection, addiction, drug abuse, or exposure to toxic matter. He mentioned that his illness was probably triggered by swimming outdoors in August 2021, and several swimming companions had fever too, but others had mild symptoms and recovered in 2 to 5 days. Physical examination revealed decreased breath sounds and audible whistles in the left lung, cervical, supraclavicular and inguinal lymph nodes were not enlarged. His chest computed tomography (CT) scan found a large hilar lesion (57 mm * 33 mm * 59 mm) combined with air bronchogram and bronchiarctia inside, multiple mediastinal lymph node enlargements (**Figure 1A**), especially subcarinal lymphadenopathy (29 mm*17 mm), local interlobular septal thickening and patchy high-density shadows in the left lung with blurred edges, multiple solid nodules in both lungs, and contrasted CT scan showed mild to moderate enhancement (**Figure 1B**). Whole body bone imaging was normal. Laboratory tests reported a leucocyte count of 19.21 × 10^9^ cells/L↑, neutrophil ratio 81.7%↑, mononuclear leucocyte 0.94×10^9^ cells/L↑, C-reactive protein 74.61 mg/l↑, serum albumin 34.3 g/L↓, serum globulin 51.6 g/L↑, and serum immunoglobulin G 34.6 g/L↑. Blood T-Spot. tuberculosis test was positive, but his purified protein derivative test was negative in local hospital. HBV surface antigen 112.70 IU/ml↑, HBV e antibody >4.50 PEIU/ml↑, HBV core antibody >45.00 PEIU/ml↑, HBV surface anti-antibody and HBV e antigen were in the reference range. The quantitative result of HBV DNA was lower than the lower limit of detection value. Serum transaminase and bilirubin were normal. The T lymphocyte count, CD4^+^ T lymphocyte count, CD8^+^ T lymphocyte count, and CD4:CD8 ratio were normal. HIV antibodies, hepatitis c virus antibodies, cryptococcal latex agglutination test (1,3)-beta-D-glucan antigen, galactomannan antigen, Aspergillus IgG antibody, carcinoembryonic antigen, neuron specific enolase, cytokeratin 19 fragment in blood were nagetive or within normal reference range. Bacterial and fungal cultures of blood and sputum were all negative. we suspected of malignant solid tumors complicated with obstructive pneumonia in the first place on admission. Of course, other diagnoses were also included in the scope of differential diagnosis, sush as tuberculosis, lymphoma, pulmonary sarcoidosis. And then he was treated with moxifloxacin and scheduled for further assessments. Electronic bronchoscopy and EBUS-TBNA of the lung and lymph nodes were conducted on Jan 22nd. Bronchoscopy revealed a slightly widened carina, stenosis of the left bronchial lumen, and infiltrative changes in the left bronchial mucosa. Ultrasound bronchoscopy found 20 mm * 20 mm hypoechoic areas in the 7th group of lymph nodes (subcarinal lymph nodes) and a hypoechoic area of 20 mm * 15 mm in the left upper lobe lung. Lymph node and lung tissue aspiration biopsies were guided by ultrasound bronchoscopy in both lesion sites. The samples were sent for cytopathology and histopathology analysis and traditional microbiological smear and culture. However, no tumor cells were found in the cytopathology and histopathology analysis, and neutrophil accumulation and abscess formation were found under the microscope, and no evidece of acid-fast bacilli was found by acid-fast staining. Since the pathogen could not be identified and cancer had not been completely excluded, the patient underwent CT-guided percutaneous puncture of the left lower lung biopsy (**Figures 1G, H**) in the later days, and seven lung tissue samples were sent for culture, mNGS, and histopathology analysis. Histopathology still showed fibrinous pneumonia, and bacterial infection was suspected, but no fungi were discovered by periodic acid-Schiff staining, hexamine silver staining, and high iron diamine/Alcian blue mucin staining. Lung tissue mNGS and culture were both negative.

**Figure 2:**
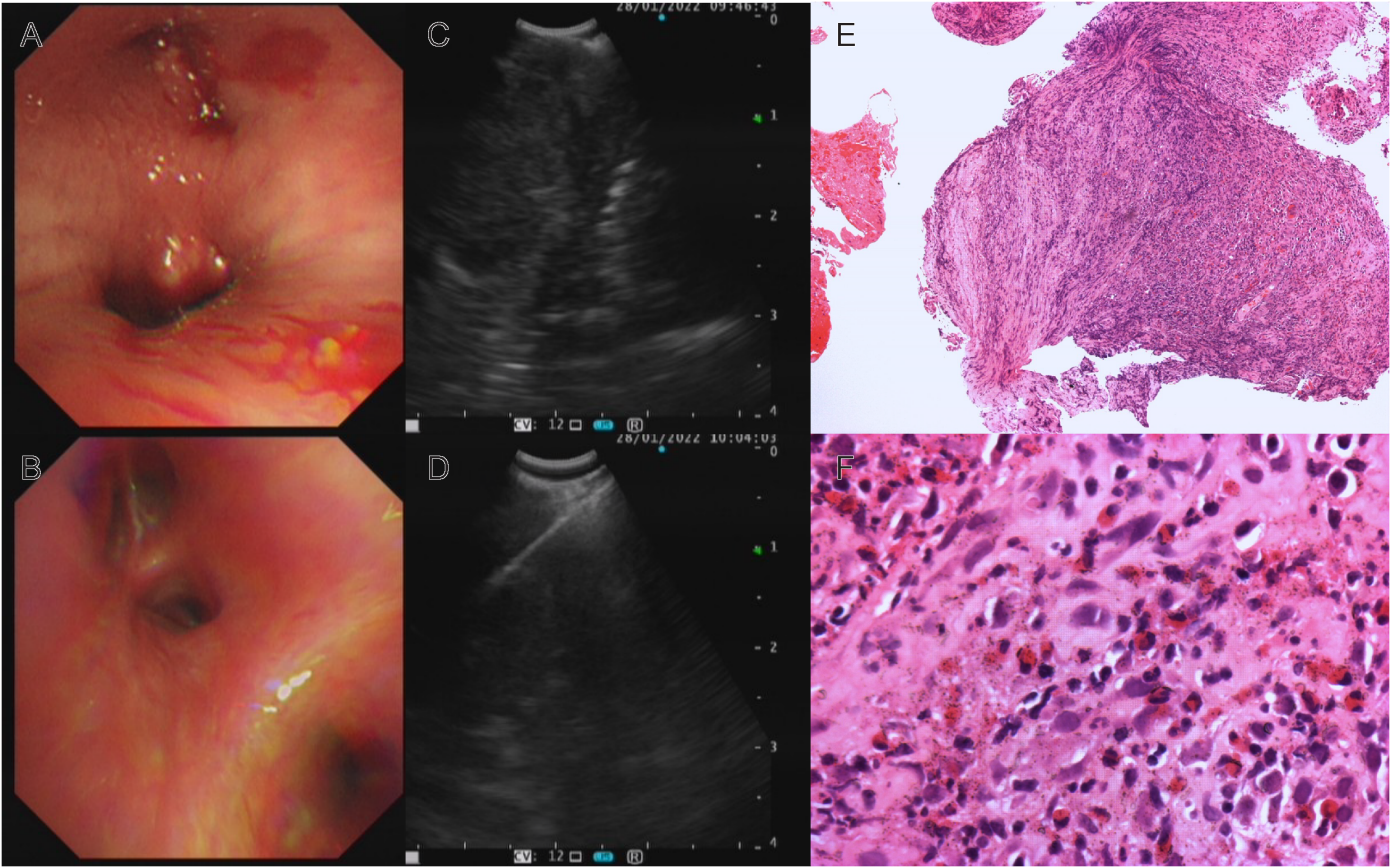
Bronchoscopy, EBUS-TBNA and histopathology: **(A)** Red and swollen infiltration of the left bronchial mucosa, aggravation of lumen stenosis, new organisms with purulent spots; **(B)** The left bronchial mucosal lesions were absorbed, the neoplasm disappeared, and the bronchial stenosis was significantly relieved after 16 days of voriconazole treatment; **(C)** EBUS showed ultrasound hypoechoic area; **(D)** EBUS-TBNA of subcarinal lymph node; Histopathology analysis: Hematoxylin-eosin staining revealed inflammatory granulation tissue hyperplasia, a large number of lymphocytes, plasma cells, and neutrophils infiltration **(E)** ×100; **(F)** ×400).

**Figure 3:**
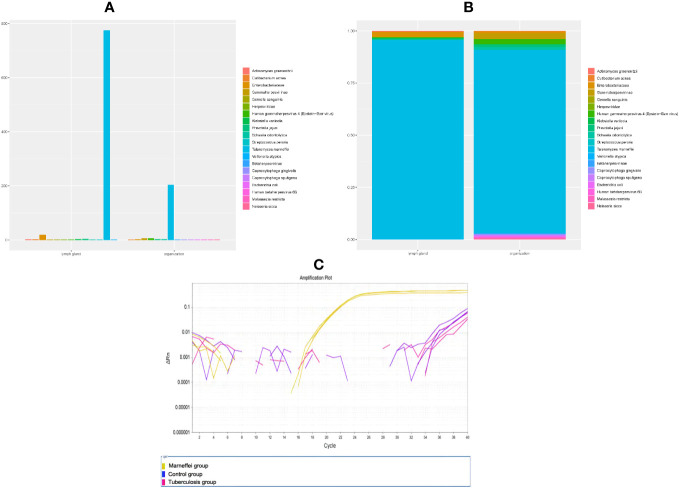
Content of *T. marneffei* in samples of each group (species and family level, **(A)** histogram, **(B)** component ratio). **(C)** QPCR amplification curve of each sample.

Thus far, the diagnosis remained unclear, and the patient has begun to experience breathing difficulties. After multidisciplinary discussions, we recommended that the patient receive EBUS-TBNA of hilar tissue and lymph node biopsy again. In addition to routine histopathology analysis and microorganism culture, specimens were strongly recommended for mNGS. On Jan 28th, his bronchoscope showed red and swollen infiltration of the left bronchial mucosa, aggravation of lumen stenosis, and periodic blockage of new organisms at the orifice of the left upper lobe with rice-grain-size purulent spots. A new peanut-sized organism was seen at the inferior lobe bronchus with purulent spots (**Figure 2A**). In addition, the bronchial stenosis of the left lower lobe was more obvious, so the bronchoscope could not reach the lower lobe. The bronchoalveolar lavage fluid was collected for microbial smears and culture, and the new organisms were taken for histopathological analysis and mNGS. A 40 mm * 30 mm ultrasonic hypoechoic region was detected by ultrasound bronchoscopy in the 7th lymph node (**Figure 2C**), and a 20 mm * 15 mm hypoechoic area was detected in the 11th lymph node of the left lung. Both ultrasonic hypoechoic regions were punctured and extracted again with the guidance of ultrasound bronchoscopy (**Figure 2D**). Lymph node puncture fluid and tissues were sent for pathological analysis, culture, and mNGS. On January 30th, mNGS reported that *Talaromyces* was detected in lymph node puncture tissue, with 783 *Talaromyces* nucleotide sequences (relative abundance: 92.45%) and 775 *T. marneffei* nucleotide sequences (relative abundance: 91.50%), (**Figure 3A**). In addition, 207 *Talaromyces* nucleotide sequences (relative abundance: 85.64%) and 204 *T. marneffei* nucleotide sequences (relative abundance: 84.30%) were identified in the mNGS of bronchial neobiological tissue (**Figure 3B**). Histopathological analysis showed inflammatory granulation hyperplasia in the lamina propria of the new organism of bronchial mucosa and infiltration of many lymphocytes, plasma cells, and neutrophils (**Figures 2E, F**). Still no fungal spores or hyphae, acid-fast bacilli or tumor cells were found in new organism of bronchial mucosa, or lymphoid tissue in periodic acid schiff staining, hexaamine silver staining, mucus carmine staining, acid-fast staining and HemateinEosin staining. Considering the notably high sequencing reads of *T. marneffei* compared to the negative control (in which no *T. marneffei* nucleotide sequence was detected), exclusion of tumor, active pulmonary and lymph node tuberculosis, and the patient’s clinical manifestation, the patient reached the diagnosis of *T. marneffei* infection of bronchopulmonary and mediastinal lymph nodes.

We supplemented quantitative polymerase chain reaction(qPCR) base on patient’s bronchial neobiological tissue. Method : Take 4~6 pieces of 5μm thick paraffin-embedded bronchial neobiological tissue sections and put them into 1.5ml EP tube, and extract tissue DNA according to the instructions of QIAamp DNA FFPE Tissue Kit (Cat.56404). QPCR reactions were performed using Vazyme 2 × Taq Master Mix (P111-01) on ABI Life7500 fluorescence quantitative PCR instrument. Experimental grouping: control group: lung tissue of patients with pneumonia caused by common bacteria was amplified by qPCR with specific primers of *Marneffei* (forward: 5’-ATCTAAATCCCTTAACGAGGAACA-3’, 5’-reverse: CCGTCAATTTCTTTAAGTTTCAGCCTT-3’); *Marneffei* group: qPCR amplification of present patient’s bronchial neobiological tissue samples with *Marneffei* primers; *Tuberculosis* group: QPCR amplification was performed with primers of *Mycobacterium tuberculosis* IS6110 gene(forward: 5’-CCTGCGAGCGTAGGCGTCGG-3’, reverse: 5’-CTCGTCCAGCGCCGCTTCGG-3’) in present patient’s bronchial neobiological tissue samples. Three duplicate wells were set in each group, and the average CT value was detected. Result: The *Marneffei* primers amplified well and reacted positively in qPCR of bronchial neobiological tissue samples (CT: 21.15~21.39), *Marneffei* primer was negative in control group samples (CT>40). While the patient’s bronchial neobiological tissue samples were amplified with mycobacterium tuberculosis IS6110 gene primers, and the reaction was negative (CT>40): The amplification curve is shown in **Figure 3C**.

Then, antifungal therapy was initiated with amphotericin B liposomes instead of antibacterial therapy. Unfortunately, the patient developed atrial fibrillation and dyspnea upon the first dose of amphotericin B liposomes, so we adjusted his antifungal therapy to intravenous infusion of voriconazole. After 4 days of antifungal treatment, the patient no longer had a fever, and the symptoms of cough and dyspnea were relieved. On the 16th day of voriconazole treatment, bronchoscopy revealed that the lesion and airway stenosis had been relieved (**Figure 2B**). The patient’s lesions were significantly absorbed on chest CT scans (**Figures 1C, D**).

**Figure 4:**
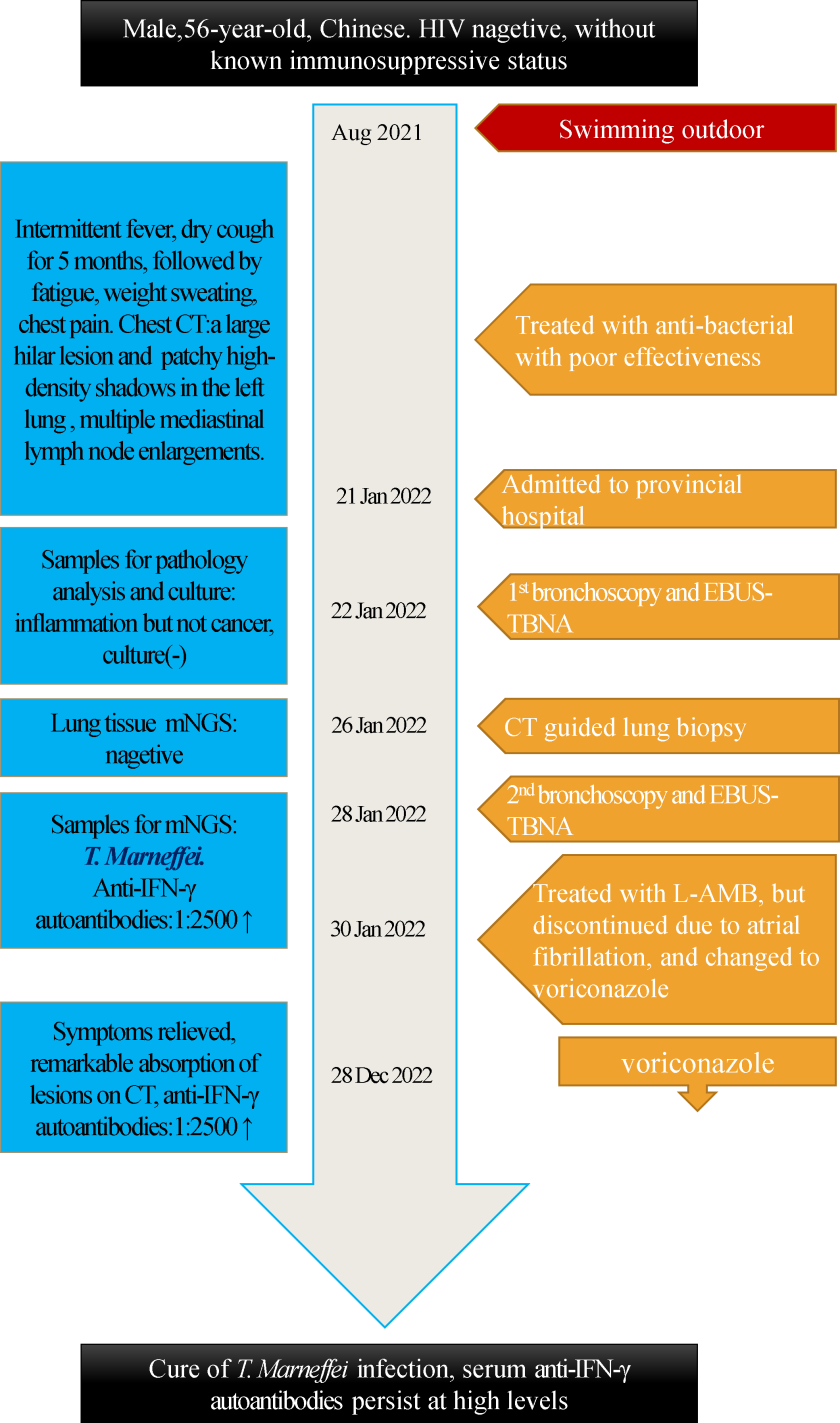
Timeline of *T. Marneffei* Infection patient.

Since there were still doubts about the actual immune status of the patient, clinicians suggested that patients be tested for anti-IFN-γ autoantibodies and whole-exome sequencing. His plasma anti-IFN-γ autoantibodies were positive with a high titer at 1:2500↑, but he refused whole-exome sequencing. The bronchoalveolar lavage fluid culture of tuberculosis was negative which was reported on 28 Mar 2022. After six months of voriconazole treatment, he recovered from the *T. marneffei* infection well, his lesions disappeared on chest CT scans (**Figures 1E, F**). But his plasma anti-IFN-γ autoantibodies remained unchanged at the sixth month of follow-up. The timeline can be seen in the **Figure 4**.

## 3 Overall discussion

This is a rare clinical case introducing the diagnosis of bronchopulmonary mediastinal lymph node *T. marneffei* infection creatively using EBUS-TBNA and mNGS techniques. In this case, the patient had a 5-month course of disease and a hilar mass with multiple mediastinal lymphadenopathies. The critical step of diagnosis is acquiring lung lesions and lymph nodes for further examination. The lesions outside the trachea or bronchus are the “blind area” of conventional bronchoscopy because they can only reach lesions in the trachea and bronchus; thus, it usually has nothing to do with lesions outside the tube (Panchabhai and Mehta, 2015). This patient underwent traditional bronchoscopy in the local hospital, but the cytopathological analysis was negative. Ultrasonic bronchoscopy is a kind of equipment that installs an ultrasonic probe in front of the bronchoscope, which can clearly show the relationship among blood vessels, lymph nodes, and occupying lesions in the extra airway mediastinum. With the guidance of color Doppler, transbronchial needle aspiration biopsy can be performed under the guidance of real-time ultrasound. The cells and tissues of the corresponding site can be obtained by EBUS-TBNA, which is accurate, safe, and efficient (Błach et al., 2021). The diagnosis of hilar lesions and mediastinal lymphadenopathy by EBUS-TBNA has been recognized widely (Błach et al., 2021). Due to the poor effect of antibacterial treatment in the chronic course of the disease and non-HIV, at the beginning, we also assumed that the patient was suffering from malignant tumors and sent the first batch of samples obtained by EBUS-TBNA to histopathological examination and traditional microbiological smear and culture. However, there were no tumor cells but only inflammatory cells detected in the tissues under pathological analysis. Since pathogens found in tissues can be used as evidence for the diagnosis of infection, the physicians discussed with pathologists and performed fungus-related staining and acid-fast staining in tissue sections but could still not find fungi or acid-fast bacilli. According to these findings, the suspected diagnosis changed from a malignant tumor to a special pathogen infection. There are many different categories of special pathogens, including tuberculosis, fungus, and virus infections, which can lead the diagnosis to many possibilities. What kind of pathogen infection was it? Traditionally and academically, a solid diagnosis of infection requires pathogenic microorganism evidence. Only one biopsy pathological result indicating inflammation cannot exclude malignant tumors or lymphoma. During the second ultrasonic bronchoscopic examination, our clinicians performed biopsy of the subcarina lymph node and left hilar lesion with EBUS-TBNA again. To obtain clear etiological evidence as soon as possible, clinicians utilized unbiased mNGS. In parallel, the samples were also sent for pathological analysis and microbiological culture. MNGS detected specific DNA fragments of *T. marneffei* in both lymph node and lung tissue specimens. Combined with the clinical manifestations of fever, dry cough, neutrophilic granulocytosis, infiltration of inflammatory cells in tissue and granulation tissue formation and qPCR, the diagnosis of bronchopulmonary mediastinal lymph node infection of *T. marneffei* was established. Therefore, we revised the patient’s diagnosis and initiated antifungal treatment.

Infection with *T. marneffei* is a rare disease in non-HIV patients, and most cases have been reported in South China. *T. marneffei* can spread through the lymphatic and blood systems, so immunocompromised patients are with higher susceptibility to disseminated *T. marneffei* infection in endemic areas (Cao et al., 2019). This patient was not infected with HIV, and there was no known immunosuppressive factor. His infection was currently limited to the lung, bronchi, and mediastinal lymph nodes, so clinicians did not consider *T. marneffei* infection before the second EBUS-TBNA. Traditionally, the microbiological diagnosis of fungal infection mainly depends on the isolation of fungal pathogens and/or direct microscopic examination of clinical specimens with the help of staining. It usually takes 1-2 weeks to culture fungal pathogens. Even if the fungus is successfully isolated, identification is challenging because the cultured microorganisms may be uncommon fungi in clinical laboratories. And pathologists may failure to identify any causative microorganisms in histological and cytological samples. The transition from Sanger sequencing of nucleic acids to next-generation sequencing technologies resulted in a rapid fall in sequencing costs approximately in 2008. Most pathogens can be detected in metagenomic next-generation sequencing (mNGS), which shows a dramatic advantage over traditional culture in pathogens that are difficult to grow or specimens with little pathogenic microorganism load (Sidiq et al., 2016). In the past decade, NGS has gone beyond lab research field and been widely utilized in clinical applications. Professor Zhang and his team first reported a case of HIV-negative disseminated Talaromyces infection assisted by mNGS in 2018 (Zhu et al., 2018). Specific DNA fragments of *T. marneffei* were detected in bone marrow, cerebrospinal fluid, bronchoalveolar lavage fluid and skin tissue (Zhu et al., 2018). In June 2021, Chi-Ching Tsang et al. reviewed the current application of NGS in diagnosing of fungal infections (Tsang et al., 2021). Since the first case was reported in 2014, more than 300 fungal infections have been diagnosed by NGS, and mNGS has been an effective method for microbiological diagnosis in pulmonary mycosis, including *T. marneffei* (Tsang et al., 2021). In our case, the pathogen was accurately and rapidly identified by mNGS and EBUS-TBNA biopsy in less than 48 hours, which was much faster than the case reported in 2018. MNGS test was faster than the pathological analysis and microbial culture. Our supplementary qPCR results showed that *Marneffei* was positive in bronchial neobiological tissue, which confirmed the report of mNGS.

It is worth noting that the patient also underwent a CT-guided percutaneous lung biopsy before the second ultrasonic bronchoscopy. Percutaneous lung biopsy is mainly used to obtain peripheral pulmonary lesions, but it is difficult to obtain samples from hilar and mediastinal lymph nodes (Mallow et al., 2019). In this case, the patient’s chest CT showed increased density in the left lower lung, and a focus in the left lower lung was obtained by percutaneous lung biopsy, but the results of lung tissue culture and mNGS were both negative. The histopathology analysis of the left lower lung indicated bacterial pneumonia, which may interfere with the final diagnosis. It was speculated that the primary disease was mainly located in the left hilum and mediastinal lymph nodes, which affected the left lower bronchial stenosis, resulting in obstructive inflammation of the left lower lung. Samples of the CT-guided percutaneous lung biopsy were aseptic inflammation, so no specific DNA fragments of microorganisms were detected by mNGS. EBUS-TBNA seems superior to percutaneous lung biopsy in hilar lesions and mediastinal lymphadenopathy.

Where did the patient’s *T. marneffei* infection come from? The natural reservoirs of *T. marneffei* in wild rodents are well-defined (Cao et al., 2019). The patient has long lived in southwestern China, where bamboo rats inhabit. His living environment was clean, without any signs of wild rodent activity. However, the patient had swum outdoors before falling ill, and several of his swimming companions had developed a fever. Therefore, it is highly suspected that the swimming pool may have been contaminated by *T. marneffei* spores from wild rodents. There was a strong possibility that the infection was caused by inhaling *T. marneffei* fungal spores through the swimming pool. Although others experienced mild fever symptoms and recovered quickly, why did the patient experience severe symptoms that tended to worsen? But the patient reported in this article was not infected with HIV, had a normal T-cell count, and had no history of recurrent respiratory infections. Reviewing the literature, we realized that although patients were not infected with HIV or were receiving immunosuppressive therapy, patients might be in a state of immunodeficiency that had not been clinically diagnosed, such as coexisting gene mutations (Ba et al., 2021; Ding et al., 2021; Shen et al., 2021) or interferon-γ immunodeficiency (Liang et al., 2020; Qiu et al., 2021). The patient in this article was with a high titer of anti-INF-γ autoantibody, which leaded to a decrease in the immune ability of interferon-γ, called adult-onset immunodeficiency syndrome. In our 2-month and 6-month follow-up, the high-titer anti-IFN- γ autoantibodies did not decrease with the control of *T. marneffei* infection, which meant that the patient’s immunosuppressive state has not been lifted. It has been reported that high-titer anti-IFN- γ autoantibodies is associated with severe intracellular infection. The patient’s positive T-spot may indicate a previous latent tuberculosis infection, but it was important to note that he have not yet received anti-tuberculosis treatment. It was necessary to be vigilant for any potential recurrence of the infection. Preventive tuberculosis intervention should be considered. Although the patient’s *T. marneffei* infection has been cured, more attention should be paid to the treatment of high-titer anti-IFN- γ autoantibodies.

## 4 Conclusions

The case of *T. marneffei* infection of bronchopulmonary mediastinal lymph nodes reported in this paper is a rare case diagnosed by mNGS combined with EBUS-TBNA among non-HIV patients, which provides a valuable reference for further research on this diagnostic approach. Complementing the traditional laboratory and bronchoscopy, mNGS combined with EBUS-TBNA may facilitate rapid and precise diagnosis of bronchopulmonary mediastinal lymph nodes fungi infection. The real immune status of “immunocompetent” *T. marneffei* infection patients requires more clinical consideration and exploration. Clinicians should be aware of anti-INF-γ autoantibodies in opportunistic infections of non-HIV patients. The high-titer anti-IFN- γ autoantibodies needs more follow-up and exploration.

## 5 Patient perspective

The main symptoms of the patient were recurrent fever and cough, that could not be relieved after antibiotic treatment, so he was worried about suffering from malignant tumor. EBUS-TBNA combined with mNGS enabled him to get a diagnosis of *T. marneffei* infection accurately and quickly. After being treated with voriconazole, his symptoms disappeared and pulmonary focus absorption was observed during follow-up. He was glad to have returned to normal work and life. He also expected better treatment for high concentrations of anti-INF-γ autoantibodies levels for preventing recurrence or suffering from other infections. The patient consented for the case report publication.

## Data availability statement

The raw data supporting the conclusions of this article will be made available by the authors, without undue reservation.

## Ethics statement

The studies involving humans were approved by Ethics Committee of The People’s Hospital of Guangxi Zhuang Autonomous Region. The studies were conducted in accordance with the local legislation and institutional requirements. The human samples used in this study were acquired from primarily isolated as part of your previous study for which ethical approval was obtained. Written informed consent for participation was not required from the participants or the participants’ legal guardians/next of kin in accordance with the national legislation and institutional requirements. Written informed consent was obtained from the individual(s) for the publication of any potentially identifiable images or data included in this article. Written informed consent was obtained from the participant/patient(s) for the publication of this case report.

## Author contributions

All authors contributed to the article and approved the submitted version. Conceptualization: GC, ZH, ZQ; Formal analysis: NM, DZ, HZ, QL, JM, YS, HL, LL; Writing-original draft preparation: GC, NM, DZ, HZ, QL, JM, YS, HL, LL. Supervision: YS, HL, ZH, ZQ; Treatment of the patient: ZQ, JM, YS, LL.
